# Combined Implant and Tooth Support: An Up-to-Date Comprehensive Overview

**DOI:** 10.1155/2017/6024565

**Published:** 2017-03-23

**Authors:** Mahmoud K. Al-Omiri, Maher Al-Masri, Mohannad M. Alhijawi, Edward Lynch

**Affiliations:** ^1^School of Dentistry, University of Jordan, Amman, Jordan; ^2^The City of London School of Dentistry, BPP University, London, UK; ^3^Faculty of Dentistry, School of Health, BPP University, London, UK; ^4^Department of Dentistry, Ministry of Health, Amman, Jordan; ^5^University of Nevada, Las Vegas, NV, USA

## Abstract

*Objectives*. This article presents a review on the concerned topics and some considerations related to the concept of splinting teeth and implants in the rehabilitation of partial edentulism.* Study Selection*. An electronic PubMed/MEDLINE and manual search of identified articles and reviews as well as clinical, laboratory, and finite element studies was performed in this project. Due to the shortage in within-subject, long term, randomized, controlled clinical trials regarding the subject a meta-analysis was not possible.* Results*. Although surrounded with some controversy, joining teeth and implants during the rehabilitation of partial edentulism provides the clinicians with more treatment options where proprioception and bone volume are maintained and distal cantilevers and free end saddles are eliminated. It makes the treatment less complex, of less cost, and more acceptable for the patient.* Conclusions*. Whenever suitable and justified, combining implant and tooth support might be recommended as an alternative during rehabilitation of partial edentulism. Based on the literature, clinical tips and suggestions were recommended to increase the success of this treatment.

## 1. Introduction

Connecting implants and teeth is sometimes considered for the support of prostheses in partial edentulism [1–124]. The published literature demonstrates the existence of considerable controversy and debate on whether it is recommendable to splint teeth to implants [1–7]. It is widely accepted that it is less than ideal to connect rigid ankylosed implants to relatively mobile dentition [8–10]. However, despite their limitations, some long term clinical studies did not demonstrate adverse effects of linking natural teeth to dental implants [11–16]. The implant-tooth supported bridges function in their biological environment without adversely affecting it [17].

Some researches [11–13, 17–22] supported linking teeth to implants mainly based on the adequate outcomes of such treatment, while others [5, 6, 8, 23–27] pointed out the importance of avoiding such paradigm when possible due to the difference in support at both ends of the system. Belser et al. [19] suggested that “a combination of implant and tooth support for fixed partial dentures is acceptable.” High levels of patients' satisfaction with implant and tooth supported fixed prosthesis were reported [20]. Also, Lindh [28] concluded that teeth should not be extracted for the sake of avoiding tooth-implant connection and that connecting teeth and implants is a practical option for supporting fixed bridges.

Many studies demonstrated no disadvantageous effect of connecting abutment teeth to implants by fixed partial dentures. Also, there are no harmful effects of this system to the opposing teeth [17]. Fixed partial dentures supported solely by implants or by teeth and implants were reported to provide fully satisfactory function and had similar high levels of predictability [29]. Furthermore, when using rigid functional connections, similar favourable values of biological and technical complications were reported when fixed partial dentures were supported solely by implants or by teeth and implants [30]. Also, similar implant survival and prosthesis success rates were found when prostheses were supported by tooth and implant or free standing implants [31].

On the other hand, some researchers showed that implants had better survival rates if they were not combined with teeth for supporting fixed partial dentures [34]. They concluded that connecting teeth and implants should only be restricted for situations including anatomical limitations, implant failure, and patient economic status and preferences [34].

Moreover, some researchers recommended avoiding combining tooth and implant support where possible and that long edentulous spans, which are not indicated for conventional fixed partial dentures, are also not suitable for combined tooth and implant support [35].

Having discussed the above debate and despite the existing controversy, this treatment paradigm seems helpful in certain situations and provides the solution to some problems (function and esthetics) and patient-centred issues when partially edentulous patients are treated using implants. It is a rational alternative in some clinical situations such as free end saddles, distraction osteogenesis, and large tissue defects. Also, it is a viable option and justified when anatomy, patient preference, and financial issues hinder the successful use of conventional treatment solely supported by teeth or free standing implants since it makes the treatment less complex and of less cost.

This article aimed at highlighting the key points related to the issue of combining teeth and implants support and presents clinicians with recommendations and suggestions that could be useful to them when they decide to adopt this treatment.

Also, this paper will explore whether this treatment option should be considered as a potential hazard for failure of the rehabilitation of partially edentulous patients.

## 2. Study Selection

A PubMed/MEDLINE electronic search (1966, September 2016) was completed and supplemented by a manual search. The bibliographies from identified articles and reviews provided an additional source to locate related articles on the topic.

Due to the shortage in within-subject, long term, randomized, controlled clinical trials regarding the subject a meta-analysis was not possible. Therefore, clinical, laboratory, and finite element studies were included. Cohort, retrospective, prospective, longitudinal, long and short term clinical studies were all included in this review.

### 2.1. Inclusion and Exclusion Criteria

English language studies, with the full text available, in core journals were included. Careful selection process, using the hierarchy mentioned above, was used as far as possible when making the final selection of the 124 [1–124] papers to be included. The terms connecting teeth to implants, tooth-implant connection, combined tooth and implant support, tooth-implant support, joining teeth and implants, splinting teeth and implants, free standing implants, rigid connection, nonrigid connection, intrusion of teeth, and combinations of the above terms were used to electronically search for articles on this topic. Duplicate studies obtained during the search and studies on removable prostheses only were excluded.

**Figure 1:**
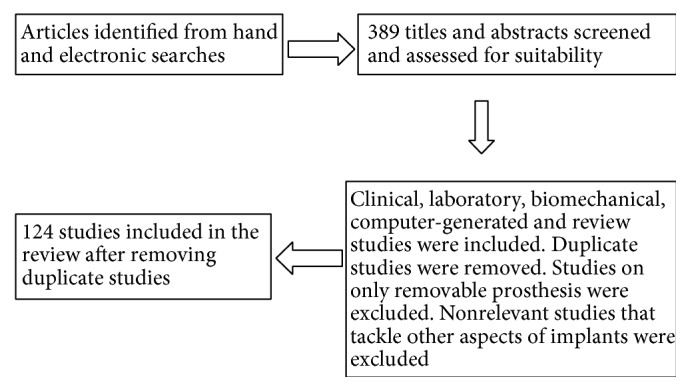
Diagram showing the process of selection of papers for this review.

Studies without English abstract were not included. All clinical, laboratory, and review articles were included.  Figure 1 presents a diagram of the involved process during selection of articles for this review.

## 3. Results and Discussion

### 3.1. Advantages of Joining Teeth and Implants

When remaining natural teeth distribution, condition, or number are not favourable for rehabilitating the mouth via fixed conventional prosthesis, teeth can be connected to implants in order to meet such a goal [4, 38]. Therefore, joining teeth and implants is recommended to be the second-choice treatment that can be used for reasons related to anatomical structures, maintaining proprioception, financial issues, and/or patient preference [4, 5, 26, 27, 29, 36–38]. Stabilization of teeth when teeth and implants are side by side is another possible advantage of joining teeth and implants [18, 32].

Previously published case reports demonstrated that this treatment can be helpful in restoring anatomy, function, phonetics, and aesthetics after oral ablative tumour surgery and bone resection in young and adult patients [39–42]. Connecting teeth and implants was also used to support distraction osteogenesis devices to allow successful augmentation of bone length and height [40, 41]. Therefore, connecting teeth and implants allowed bridging large bony defects without the need for bone augmentation mesially or distally of a tooth, and this minimizes surgical risks, treatment time, and treatment costs.

In summary, joining an implant to a tooth can be an alternative in some clinical indications such as free end saddles and large tissue defects, which provides solution to some anatomical, functional, or aesthetic problems. Also, it can be a practical option in certain conditions where patient preference and financial issues hinder the successful use of conventional treatment solely supported by teeth or free standing implants.

### 3.2. Occlusal Load Absorption in Teeth-Implant Connections

Teeth mobility might be 10 times that of a dental implant [43]. Connecting teeth to implants results in a challenging biomechanical system that provides the prosthesis with different support, and thus stress, at both ends of the system [44–47]. The implants are ankylosed and prevent tooth loading; therefore, teeth might contribute little support in this situation and become infraerupted or affected by tooth intrusion [1, 5, 23, 45, 47–53]. This also leads to implant overloading and makes it difficult to obtain ideal occlusion and thus undermines the use of such treatment option [8, 54]. In addition, a cantilever effect off the implant supported portion of the system might result and cause high bending moment under loading and thus result in loss of osseointegration, fracture of the prosthesis, or abutment screw loosening [2, 7, 20, 36, 64–66]. Therefore, to minimize the occlusal force on a pontic by occlusal adjustments to maximize stress distribution in centric position and lateral movements is recommended [46, 67–69].

Nevertheless, such concerns were not supported by many in vivo and in vitro studies which demonstrated that teeth and periodontium shared the support and this share increased with the amount of load [11, 12, 36, 55–63].

Many FEA and photoelastic stress analysis studies were conducted to study the stress distribution within the system of connected teeth and implants. Some researchers found that implant and alveolar bone stresses were not dependent on the type of the connection [68, 69]. However, they demonstrated that the stress within the prosthesis was doubled [68] and increased more than 3-fold [69] when nonrigid connector was used in comparison to rigid one.

In contrast, other researchers [21, 70–73] found that stresses were mainly concentrated in and around the implant when it was connected to a tooth. Consequently, increasing the number of fixtures rather than abutment teeth, placing minimal loading on teeth, and directing most of the load to implants in order to optimize the stress distribution within the system were recommended. Also, some researcher [72, 73] recommended placing a nonrigid connector on the implant abutment-supported site to reduce such stress. Although nonrigid connectors are more effective in compensation for the difference in mobility between teeth and implants under axial loading, unfortunately, this will be on the expense of the stress distribution within the prosthesis which in turn will be increased [68, 69].

It was found that, under vertical loading, placing nonrigid connector over an implant would reduce bone stresses formed around implant [74]. Using the patrix of the NRC on the implant may more effectively reduce stress formation around the implant.

Although FEA studies support the presence of differential support between teeth and implants, in vivo and mechanical in vitro studies reported no such tendency and demonstrated no major differences in support on both ends of the system regardless of bone or prosthesis flexibility [36, 57, 63]. This was attributed to the inherent flexibility of the implant system where the screw joint forms a flexible system. Moreover, the implant provides most of the support under light loading but when loading exceeds 10 N the tooth begins to share load and contributes to support of the prosthesis [63]. Also, distribution of the load between abutments was influenced more by prosthesis geometry and implant placement [57].

Using IMZ implants, a retrospective radiographic study supported with a mathematical model concluded that bone loss and atrophy were increased when teeth were connected to implants at a distance of 8–14 mm between the first implant and the abutment tooth and a distance of 17–21 mm between the second implant and the abutment tooth [75]. However, the IMZ implants have a well-documented history of progressive bone loss even in cases where the prosthesis is solely supported with implants. Therefore, the data from this study may be relevant for the IMZ implants only.

Also, some researchers recommended that the fixed partial denture span should not exceed three units when tooth and implant support was used [76].

Some researchers demonstrated that using the retaining-screw 3-piece implant connected to an abutment was associated with better stress distribution and was a more viable option than using a taper integrated screw-in 2-piece implant when an endodontically treated tooth was connected to an implant for fixed partial denture support [77]. They also demonstrated that repeated load fatigue was an important cause for tooth-implant support system failure [77].

### 3.3. Considerations before Joining Teeth and Implants

Some factors should be considered before joining teeth and implants including prosthetic design and occlusion, condition of tooth (periodontal stability, caries, endodontic problems, angulation, and position), parafunctional activity, and patients' expectations and motivation [14, 28, 33, 44, 78].

More failures of this treatment modality were associated with maxillary bone, short implants, tilted implants, poor bone quality, and using endodontically treated teeth as abutments [14, 20, 28, 50, 51, 81, 82]. Block et al. [51] reported the removal of 5 abutment teeth that were endodontically treated and connected to implants after being fractured at the interface of the post within the tooth. Lin et al. [46] concluded that connecting teeth and implants in regions with reduced bone quality was associated with significantly increased bone stress levels. However, stress levels within teeth or implants were not affected with the bone quality, while reducing the load on pontics significantly decreased stress within the implant, the tooth, and the alveolar bone. Furthermore, a long term follow-up clinical study of implant and tooth supported prostheses reported more biological complications and failures when abutment teeth were endodontically treated or had reduced bone attachment levels (i.e., affected by periodontal disease) [15]. Also, tilting the implants has biomechanical effects including increased peri-implant strains within implant-tooth supported prostheses during torque-tightening and under load [81].

Most failures were found related to loss of osseointegration, periapical tooth infection, tooth intrusion, ceramic fracture, and screw loosening [33, 59]. The intrusion phenomenon (5%) was only demonstrated when the nonrigid connections were used [33, 59]. Therefore, to use rigid connection to avoid intrusion of the abutment tooth was recommended.

To use sound abutments, a bicuspid-wide pontic length, and rigid connectors when joining teeth and implants is planned is recommended [14].

In summary, adequate tooth condition, implant inclination, implant size, occlusion, prosthetic design, prosthesis span length, periodontal health, and bone quality and quantity are essential for improving the success rate of this treatment.

### 3.4. Periodontal Ramifications of Joined Implants and Teeth

Studies on animals reported similar periodontal breakdown around teeth regardless of being splinted to implants or not [61, 83, 84]. Pesun et al. [85] found that histology of periodontal ligament around teeth connected to implants was similar to control teeth with minimal inflammation and minimal remodeling. The crestal bone was cortical and showed no breakdown of the periodontium while the blood vessels morphology was similar to the control teeth. Such findings led to the conclusion that splinting teeth to implants does not negatively affect the periodontium which has suitable remodeling capacities to overcome the load. Another histological and clinical study on 8 partially edentulous monkeys reported no clinical difference between different prosthetic rehabilitations supported by single freestanding implant, connected freestanding implants, or implant and tooth [86]. Histologically, direct bone deposition occurred in all groups and bone contact ratio ranged between 66% and 81%.

Clinical, microbiological, and histological studies on humans with implant and tooth supported prostheses reported that the surrounding soft tissues around both tooth and implant demonstrated favourable histological findings with minimal if any inflammatory cell infiltrates and good bone-implant contact [20, 87]. Bacterial morphotypes in plaque had similar distribution around teeth and implants and the microflora was predominated by nonmotile rods while the spirochetes were minimal or absent [87]. Moreover, changes in plaque accumulation, bleeding on probing, pocket depths, and marginal bone level were acceptable and treatable when joining teeth to implants [20].

A prospective 3-year clinical study reported increased plaque scores at implant sites, increased probing depths at implants and teeth, no significant changes in bone levels around teeth or implants, and no tooth intrusion [66]. The restorations were fully functional and successful. Also, similar success rates, mobility, bone loss, and gingival health were reported when natural teeth were compared to implants in tooth-implant supported cases [36]. However, the implants had deeper pocket probing depth (2.3 ± 0.5 mm for teeth and 3.3 ± 0.7 mm for implants). A 5-year prospective study found no adverse effects on teeth when they were rigidly splinted to Branemark system implants (Nobel Biocare, Goteborg, Sweden) [94].

The above discussion is in favour of opinions which demonstrate that there are no significant periodontal changes between the freestanding implants and those connected to teeth. Plaque accumulation, bleeding on probing, pocket depths, and marginal bone level were within acceptable and treatable limits when joining teeth to implants.

### 3.5. Survival Rates of Joined Implants and Teeth

Variable treatment success rates of implants, teeth, and prostheses were reported in the literature when teeth were connected to implants. The 5–10-year survival rates of implants joined to teeth ranged between 82% and 100% [20, 33, 34, 88]. In addition, the 2–10-year survival rates of prosthesis supported by implants and teeth ranged between 77.8% and 100% [33, 88]. Also, the 2–10-year survival rates of teeth joined to implants ranged between 90% and 100% [33, 88]. Also, implant survival rates were found to be 91% and 95.5% in the maxilla and the mandible, respectively [20].

Some studies reported similar survival rates of bridges supported with either free standing implants or implants connected to teeth [11, 12, 16, 60, 89]. Also, similar survival rates were reported for implants joined with teeth and free standing implants [11, 13, 55, 90]. Moreover, some researchers reported more marginal bone loss around implants that were not linked to teeth [60]. Hosny et al. [13] reported similar levels of bone loss, 1.08 mm for the first 6 months and 0.015 mm annually, around implants regardless of being connected to teeth or not and regardless of the number of connected teeth or implants.

Previous studies [11–13, 55, 60, 89, 90] that reported similar survival rates of free standing implants and implants connected to teeth were prospective randomized or split mouth designed (within-subject comparison studies) and used identical implant systems and treatment procedures and prostheses, and this made their results more convincing. However, some of these studies were short term [55, 60, 89] and not randomized and suffered small sample sizes (the number of patients was 23 and 18, resp., for Gunne et al. [12] and Hosny et al. [13] studies), and their results could not be generalized. Further longitudinal long term randomized studies with large sample sizes are required in this regard.

However, other studies reported better survival rates of bridges supported with free standing implants in comparison to those supported with implants joined to teeth [26, 50, 88, 95]. Moreover, some researchers found better survival rates of free standing implants in comparison to implants connected to teeth [50, 82, 88]. Previous studies [50, 82, 88, 95] that reported better survival rates of free standing implants than implants connected to teeth did not use intraindividual control [50, 82] and used different patients as controls. Moreover, most implants were in the maxilla (83.7%) which might have increased the failure rate of the implants in some studies [50]. Also, some studies [95] were in vitro and used the worst case scenario for loading and tested ceramic implant abutments rather than metal ones. Also, some researchers [26, 88] pooled all studies on fixed prosthesis with different implant systems, different prosthesis designs, and different number of restorations. Nevertheless, some of these studies [50, 82] had longer time and larger sample size than many previous studies.

It is worth mentioning that, in clinical research, the paired design of intraindividual comparisons is highly powerful in detecting differences in smaller samples; the importance of this is obviously demonstrated when the studied factor is quite variable from one subject to another [96]. The results of many studies cannot be generalized because they suffered reduced sample size, short duration, and poor designs such as no randomization and no control to free standing implants, and no within-subject control was attempted.

Clinical studies also explored the effect of the type of connection on survival rate of this treatment. Some researchers concluded that using rigid connections when joining implants and teeth would result in similar survival rates of implants and prosthesis as those achieved when prostheses were solely implant supported [30, 59, 92]. Most failures were related to loss of osseointegration, periapical tooth infection, tooth intrusion, ceramic fracture, and screw loosening [33, 59]. Also, some researchers found no effect of the type of implant surface on frequencies of complications and patterns of failure when implants were connected to teeth [97].

### 3.6. Tooth Intrusion and Type of Connector That Should Be Used

Although many theories have been proposed to explain tooth intrusion, the cause of tooth intrusion remains unclear [1, 23, 37, 43, 48, 50, 98–100]. Tooth intrusion is a multifactorial condition and might be due to disuse atrophy [50], mechanical binding [23, 37, 43, 48], mandibular flexion and torsion, flexion of the fixed partial denture, impaired rebound memory and significant energy dissipation by the elastic and inelastic deformation of periodontal ligament [23, 49], and impaction of debris and parafunctional activity [1, 23, 37, 48, 98–100].

Tooth intrusion occurs within the first year after splinting the teeth to implants but not within the first 3 months after splinting [7, 23, 48, 49]. It is a 1-time event, without progression over time [51]. The literature demonstrated high discrepancy in the occurrence of intrusion that ranged from 3% to 37% [33, 48, 51, 52, 88, 101]. This might be due to variable study designs, small sample sizes, using different connectors, and using different implant designs. However, the rate of 3.5–5% seems the most reported figure in many studies [50–101].

Without the provision of scientific evidence or well-controlled studies, some suggestions were introduced via the literature in order to overcome the problem of differential support at both ends of the system. These include bone flexibility [102], compensatory micromotion within the implant system [36, 63], introduction of IMZ implants [24, 103], using permanent cement [20, 44], using mechanical locking device (screw attachment) [7, 20, 24, 44, 105, 106], or using nonrigid connection between teeth and implants [6, 23, 25, 47, 67, 72, 107–110].

A clinical trial over 16.5 months revealed no difference in implant and tooth mobility, pocket probing depth, and bone loss when the suprastructure had fixed or mobile bedding on the IMZ implants splinted to natural dentition [104]. Less cervical stress around the IMZ implant was reported when IMC resilient element was used instead of a titanium rigid element in combined tooth-IMZ implant support [53]. Nevertheless, tooth intrusion was documented when IMZ implants were linked to natural teeth [101].

Also, Weber and Sukotjo [31] concluded that success and survival rates of implants and prostheses were similar when either screw retention or cementation was used for retention.

The issue of the relation between type of connection and tooth intrusion is controversial. Some FEA and photoelastic stress analysis studies showed that nonrigid connection was associated with less bone stress around implants but more stress within implants and prosthesis [67, 72, 80, 110, 115]. Rigid connection was associated with more bone stress around implants [67, 72, 80, 110, 115]. Also, more stress within the implant was found when the occlusal loads acted on the natural tooth in rigid connection [67]. However, regardless of the type of the connection, the stress within the implant system was not significantly increased when the occlusal loads acted on the implant, the pontic, or the entire prosthesis.

On the other hand, other FEA studies found that implant and alveolar bone stresses were not dependent on the type of the connection [68, 69]. However, they demonstrated that the stress within the prosthesis was doubled [68] and increased more than 3-fold [69] when nonrigid connector was used in comparison to rigid one. Also, the use of external hexagon implants and rigid/semirigid connection design were suggested to reduce stresses in the abutment structures when combining tooth and implant support [116].

The nonrigid connector is more effective in compensation for the difference in mobility between the teeth and implants under axial loading [68, 69, 72]. Unfortunately, this will be on the expense of the stress distribution within the prosthesis which in turn will be increased [68, 69].

The controversy among FEA studies is obvious and this could be related to the difference in the model design, using 2- or 3-dimensional models, and assumptions of the material properties especially bone elastic properties and periodontal ligament properties.

Some clinical studies [51, 52, 59, 101] demonstrated no difference in tooth intrusion between rigid and nonrigid connection designs. Other researchers found that tooth intrusion was mainly associated with normal periodontal support and nonrigid connectors while no intrusion was found in patients with reduced periodontal support regardless of the connector type [32]. In contrast, periodontal support was found to have only minor effect on stress values in implant-tooth supported systems [71].

Other researchers found that nonrigid connection design is associated with more tooth intrusion and that rigid connection is better to use [2, 9, 10, 14, 20, 30, 33, 44, 48, 50–52, 59, 88, 117]. In a meta-analysis of 13 previous studies, Lang et al. [88] found that 5.2% of abutment teeth were affected by intrusion and this was almost always occurred when nonrigid connections were used. Some researchers [51] found that tooth intrusion affected 66% of the nonrigid group and 44% of the rigid group; 25% of the nonrigid teeth had greater than 0.5 mm intrusion, compared with 12.5% for the rigid group. The high rate of intrusion in the rigid connection group could be due to the design of the prosthesis or the type of the cement used. Nevertheless, there was no significant increase in intrusion over time in any case. Furthermore, nonrigid connection would cost more and require more maintenance and visits. Others reported implant fracture due to nonrigid connection [21].

Some studies reported more bone loss (0.7 mm) around implants when rigid connection was used in comparison to nonrigid ones due to increased bending loads [50, 113]. However, these studies were not well controlled within the study population and reported various clinical situations and thus their results cannot be generalized [51]. Moreover, the patients group suffered 0.7 mm bone loss around implants that were rigidly connected to teeth while the control subjects suffered 0.4 mm bone loss. The extra 0.3 mm bone loss occurred over a 15-year period of time and would not affect the success of the prosthesis.

Some researchers [30] concluded that using rigid connection will result in similar survival rates as those achieved when prosthesis is solely implant supported. Also, rigid connection and fixation technique (screwed versus cemented) was not significantly related to the rate of technical complications [30]. Others suggested the use of rigid connection and strong cement to improve the success of implant-tooth supported prosthesis [14]. However, in an in vitro study, Mathews et al. [117] concluded that rigid or nonrigid connector design had no effect on cement retention in implant-tooth supported fixed partial denture. Moreover, some researchers suggested placing a metal coping over the abutment tooth without the need for cement between the prosthesis and the coping [108, 109]. However, abutment intrusion might be a problem and a cement might be required [98, 118].

Permanent cements might prevent intrusion by counteracting the forces that tend to cause tooth intrusion, but once the intrusion forces exceed the cement retentive forces, the tooth will be intruded and the tooth surface will be exposed to the oral environment. In comparison to tooth supported bridges, more marginal gap was detected when tooth-implant supported bridges were cemented regardless of the type of cement (resin, glass ionomer, or zinc phosphate cements) [119]. However, no significant changes in the accuracy of the prosthesis margin were identified after the prostheses were exposed to simulated oral stress in artificial oral settings. The literature lacks long term controlled studies of the effects of tooth intrusion in such situations.

The issue of joining teeth to implants using nonrigid or rigid connectors is still incompletely resolved and most published literature in this regard is uncontrolled clinical studies or clinical case reports without data analysis for accurate comparison between the two designs of connection [19, 51]. However, most of the recent studies concluded that rigid connections are superior to nonrigid ones [33, 120, 121]. Also, even if slightly more bone resorption occurred when rigid connection is used the clinical advantage of rigid connection, that is, avoiding tooth intrusion, justifies using the rigid connections rather than the nonrigid ones [33, 113].

Nevertheless, satisfaction with implant and tooth supported prosthesis was demonstrated to be very high regardless of the type of connection design [51].

The long term success of tooth-implant supported system is mainly affected by the biomechanics of the system [67, 122, 123]. Moreover, the occlusal force is the main factor that affects stress distribution within the system [67, 69, 122, 123], and the relationship between the occlusal load and the type of the connection is not completely clear [67]. Clinical and experimental studies cannot uncover such relationship and cannot identify the biomechanics of such complicated system. Therefore, finite element analysis (FEA) studies might be the answer where stress distribution and occlusal forces can be studied under controllable conditions [124]. Unfortunately, FEA studies have some shortcomings (such as difference in model designs, using 2- or 3-dimensional models, and various assumptions of material properties especially bone elastic properties and periodontal ligament properties) that might undermine their role in this regard.

Alternative treatments to splinting teeth to implants include removable partial dentures, cantilevered fixed partial dentures, overdentures, acceptance of shortened dental arch, and surgical correction of the implant receiving site to increase the number of inserted implants; for example, nerve repositioning, bone grafting, and sinus lifting can be the alternative solution to problem.

## 4. Conclusions

The subject of connecting teeth to implants is controversial. The following conclusions and recommendations are suggested:

- The first-line therapy seems to be using free standing implants for supporting fixed dental prostheses whenever possible. The most up-to-date publications show a higher need for maintenance and repair when teeth and implants were connected in comparison to free standing implant support. However, the literature presents three main schools of thoughts in this regard; one school advocates nonrigid tooth and implant connection; another prefers rigid connection, while the third recommends that implants and teeth should not be connected.
- Joining teeth and implants during the rehabilitation of partial edentulism is indicated to provide clinicians with more treatment options where proprioception and bone volume are maintained and distal cantilevers and free end saddles are eliminated. Whenever suitable and justified, such treatment option becomes a valid alternative especially if it makes the treatment less complex, of less cost, and more acceptable for the patient.
- This treatment paradigm is associated with some risks and complications including loss of osseointegration, periapical tooth infection, tooth intrusion, ceramic fracture, prostheses decementation, and screw loosening. In order to improve treatment success rate, it is better to avoid using short implants, poor bone quality, and endodontically treated teeth when this treatment paradigm is considered. Also, using rigid connection and permanent cementation are associated with less tooth intrusion and less complications.
- Further research is still required on many aspects of this treatment paradigm. No conclusive studies are available to show the best number of implants and teeth to be connected using this treatment option. In addition, no conclusive evidence is available to show the best prosthesis span length that can be supported via connecting teeth and implants. Also, studies on patient and clinician satisfaction with such treatment paradigm are not available. The literature also lacks studies regarding detailed periodontal, microbiological, and immunological studies in this field. Moreover, most studies were conducted using limited number of implant systems and valid comparisons to other systems or different implant surfaces are not available. Finally, most previous studies are not randomized and suffer from small sample sizes. Therefore, further long term randomized clinical studies with large sample sizes are required.
